# Microwave spin resonance investigation on the effect of the post-processing annealing of CoFe_2_O_4_ nanoparticles

**DOI:** 10.1039/d0na00156b

**Published:** 2020-04-06

**Authors:** Prashant Kumar, Saurabh Pathak, Arjun Singh, H. Khanduri, G. A. Basheed, Lan Wang, R. P. Pant

**Affiliations:** CSIR-National Physical Laboratory New Delhi India-110012 rppant@nplindia.org prashantkhichi92@gmail.com; Academy of Scientific and Innovative Research, CSIR-NPL Campus New Delhi India-110012; School of Science, RMIT University Melbourne VIC 3000 Australia; School of Engineering, RMIT University VIC 3000 Australia; Department of Mechanical Engineering, University of Melbourne Parkville VIC 3010 Australia; Department of Physics, Indian Institution of Technology Jammu-181221 India

## Abstract

A novel investigation on the finite-size effects on the spin resonance properties of cobalt ferrite (CoFe_2_O_4_) nanoparticles has been performed using a room temperature ferromagnetic resonance (FMR) technique. A single broad spectrum was obtained for the CoFe_2_O_4_ nanoparticle samples, which indicated that all the samples were showing ferromagnetic characteristics. An asymmetric FMR line shape with a hefty trailing section was obtained due to the high magneto-crystalline anisotropy in CoFe_2_O_4_ nanoparticles, which changed with the size distribution. The resonance field for the samples shifted to a higher value due to the increase in the magneto-crystalline anisotropy in the CoFe_2_O_4_ nanoparticles with an increase in size. A systematic change in the resonance field and line width was observed with the change in the size distribution of the particles. Initially, it decreased with an increase in the size of the particles and increased after the critical size range. The critical size range is the imprint of the shift of the magnetic domain from a single domain to multi domain. The line width increased at higher annealing temperatures due to the enhancement in the dipole–dipole interaction, which led to a higher spin concentration as well as magneto-crystalline anisotropy. Furthermore, the saturation magnetization (*M*_s_) as well as ‘*M*_r_/*M*_s_’ increased from 37.7 to 71.4 emu g^−1^ and 0.06 to 0.31, respectively. The highest coercivity (750.9 Oe) and anisotropy constant (4.62 × 10^4^ erg cm^−3^) were found for the sample annealed at 700 °C, which can be corroborated by the literature as the critical annealing temperature at which CoFe_2_O_4_ nanoparticles shift from single domain nanoparticles to multi-domain nanoparticles. Post-processing annealing is critical in advanced processing techniques and spin dynamics plays a vital role in various interdisciplinary areas of applications.

## Abstract 2

A novel investigation on the finite-size effects on the spin resonance properties of cobalt ferrite (CoFe_2_O_4_) nanoparticles has been performed using a room temperature ferromagnetic resonance (FMR) technique.

## Graphical abstract

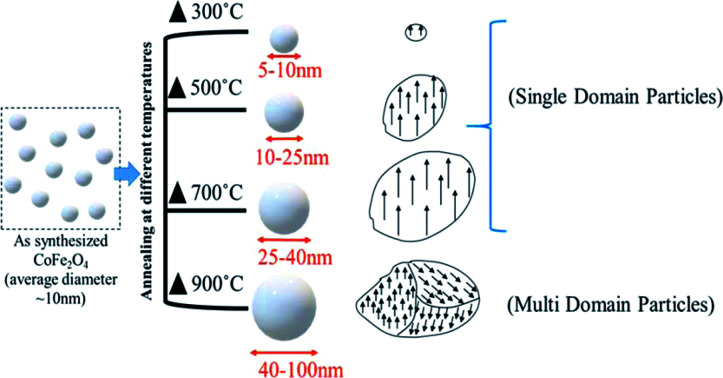

## 1. Introduction

In recent years, the evolution of electromagnetic radiation sources has undergone exponential advancement that is reflected in the increase in electromagnetic interference (EMI) shielding research, which is one of the key applications of magnetic nanoferrites.^1–5^ The nanocrystalline ferrites have been a subject area of immense potential due to their profound applications in a wide range of interdisciplinary areas such as magnetic resonance imaging, drug delivery, magnetic hyperthermia treatment, spintronics, high-density memory devices, microwave devices, and power transformers.^2,6–10^ To achieve a higher figure of merit in these applications, the properties of magnetic nanoparticles (MNPs) need to be optimized for relevant applications.

Ferrites belonging to the AB_2_O_4_ group (family) are mainly controlled by divalent cations, which occupy the tetrahedral ‘A’ sites, and trivalent cations, which have a high degree of affinity for the octahedral ‘B’ sites.^11,12^ Therefore, the whole range of distribution of cations possible in spinel ferrites can be shown by the general formula (M_1−*α*_^2+^ Fe_*α*_^3+^) [M_*α*_^2+^ Fe_2−*α*_^3+^], where cations inside the round and square brackets occupy the tetrahedral and octahedral sites, respectively. Alpha (*α*) represents the degree of inversion and shows the fraction of the tetrahedral site occupied by Fe^3+^ cations, which depends on the synthesis method and post-annealing. If *α* = 0, the spinel ferrite is normal; when *α* = 1, it is an inverse spinel ferrite. However, if 0 < *α* < 1, the spinel ferrites are mixed.^7^ The presence of two transition metal ions at the crystallographic site leads to a unique type of hopping conductivity and magnetic coupling. CoFe_2_O_4_ is an interesting material because of its high magneto-crystalline anisotropy, coercivity, chemical stability, good electrical insulation, mechanical hardness and moderate magnetization at room temperature.^12,13^ Apart from this, CoFe_2_O_4_ has excellent physical and chemical stability, which makes it suitable for magnetic recording applications as high-density digital recording disks. A recent investigation has shown that the cobalt ferrite nanoparticles have photo-induced magnetic properties.^14^ Magneto-crystalline anisotropy is a very important parameter in connection with the characterization of the materials utilized in technical applications, particularly magnetic recording media. Magneto-crystalline anisotropy strongly depends on the interaction of cations in the A and B sites, which can also be altered by changing the lattice-occupancy state, crystallite size, bond angle and bond length.^7^

Pure and doped CoFe_2_O_4_ show a variety of novel properties that vary with the nature of the cations, their charge and site occupancy distribution between tetrahedral and octahedral sites.^7,11,15,16^ Different experimental methods have been used in the preparation of ferrite nanoparticles such as sol–gel techniques,^13^ co-precipitation,^1,17^ hydrothermal,^18^ microemulsion^19^ and combustion methods,^20^*etc.* Among all of these, the co-precipitation method has been widely used as the prepared sample exhibits high crystallinity, homogeneity and good textural properties of the obtained material.^1,7,17^ Also, this technique offers an advantage due to its low cost, high-quality production and less time consumption.

Also, the magnetic properties of the CoFe_2_O_4_ nanocrystals depend on the different sizes, shapes and synthesis methods.^20,21^ C. H. Chia *et al.* prepared a series of CoFe_2_O_4_ nanocrystalline powders with varying synthesis temperatures and further annealing was done at different temperatures to optimize the structural and magnetic properties using the chemical co-precipitation method.^17^ They reported a significant increase in the saturation magnetization (*M*_s_) from 43.2 to 60.7 emu g^−1^, with the annealing temperature from 300 to 1200 °C. A similar trend was also found in the coercive field (*H*_c_), which increased from 376 Oe at 300 °C to 1201 Oe at 1200 °C. However, the coercive field *H*_c_ reached its maximum of 1373 Oe at 400 °C and then decreased to 870 Oe at 600 °C.^22^ The drastic change in *H*_c_ for high-temperature annealed samples (900 °C) might originate from the transition of a single magnetic domain to a multi-domain within a particle. Further, K. Rana *et al.*^12^ synthesized CoFe_2_O_4_ magnetic nanoparticles using the citrate precursor method and post-processing by annealing was done in the temperature range 700–1100 °C. They suggested that the increase in the crystallite size and magnetization was due to the increase in the annealing temperature. Also, the *M*_s_ and coercive field increased from 53.69 to 74.46 emu g^−1^ and 257.40 to 304.99 Oe, respectively. A linear increase in the saturation magnetization (*M*_s_) with the annealing temperature was observed in CoFe_2_O_4_ MNPs, although the *M*_s_ values of the samples were significantly lower than the bulk value of 90 emu g^−1^.^12^

Even though numerous methods have been reported extensively for the CoFe_2_O_4_ preparation, the precise control of the size and morphology to achieve desired properties is still in progress. The investigation of spin dynamics plays a key role in the EMI shielding operation and has been reported for the CoFe_2_O_4_ by many researchers. However, most of the research reports limit their discussion to the resonance field and *g*-value, which are major imprints of the material characteristics.^7,15,23^ Other key spin resonance parameters that can provide a more detailed understanding of the properties of materials required to enhance the performance of the device have rarely been reported in the literature.^7^

In the present work, CoFe_2_O_4_ MNPs were synthesized by a wet chemical co-precipitation method and post-processing was carried out by annealing the as-prepared CoFe_2_O_4_ powder at different temperatures (300–900 °C). A detailed investigation of the microwave resonance behaviour was carried out by a ferromagnetic resonance (FMR) study and the spin resonance parameters were calculated by fitting the FMR spectra. The present study emphasizes the modification of the structural, morphological, static and dynamic magnetic properties of the single-phase cobalt nanoferrites by post-process annealing.

## 2. Experimental

### 2.1 Synthesis

CoFe_2_O_4_ ferrite nanoparticles were synthesized by a wet chemical co-precipitation method having the stoichiometric ratio of Co/Fe equal to 1 : 2. High-purity chemical precursors FeCl_2_·4H_2_O, CoCl_2_·4H_2_O and NaOH (99.99%, from Sigma Aldrich) were used without further purification. The 0.23 M iron chloride (FeCl_2_·4H_2_O) and 0.16 M cobalt chloride (CoCl_2_·4H_2_O) solutions were prepared in DI water and heated to 80 °C under constant stirring for 2 hours; the pH was maintained at 10 using 8 M ammonium hydroxide (28%) solution. The precipitated particles were centrifuged at 5000 rpm followed by washing several times with ethanol and DI water to obtain a uniform and narrow size distribution of particles. The obtained precipitate was then filtered and dried overnight in the oven at 70 °C to remove the water content. Finally, the black powders of CoFe_2_O_4_ were ground into fine powder and annealed at four different temperatures: 300 °C (sample-a), 500 °C (sample-b), 700 °C (sample-c), 900 °C (sample-d) in an argon (Ar) gas atmosphere for 3 hours with a slow heating rate of 5 °C min^−1^ and then cooled down to room temperature under the same conditions. The final obtained annealed black powders were used for the different characterizations to study the annealing effects on the material properties.

### 2.2 Characterization

The X-ray diffraction (XRD) patterns of the powder samples of magnetic nanoparticles were obtained with a multipurpose X-ray diffractometer (Rigaku Ultima-IV). The diffraction pattern was recorded at a tube voltage and current of 40 kV and 30 mA, respectively, using Cu-Kα (*λ* = 1.54056 Å) radiation with the scan step size of 0.02° and a scan speed of 2° min^−1^ in the 2*θ* range from 20° to 80°. The average crystalline size was estimated by the Debye–Scherrer equation and the Williamson–Hall method. The X-ray diffraction (XRD) patterns of the samples were probed by employing the Rietveld refinement technique using the Full Prof software, which is a well-established technique for evaluating the structural parameters from powder diffraction data. The Rietveld method is a well-established technique for extracting structural details from powder diffraction data. The method employed a least-squares procedure to compare the Bragg intensities and those calculated from a possible structural model. The pattern refinement was achieved in the following two steps: the background removal and scaling by the evaluation of structural parameters and site occupancies. The morphologies of the samples were studied using a high-resolution transmission electron microscope (HRTEM), model TECNAI F30. The specimens were prepared by sonicating the powdered sample dispersed in ethanol for about 30 min, followed by a uniform disperse solution dropped on the 300 mesh copper grid and dried at room temperature. Further, the size distribution of the samples was studied using small-angle X-ray scattering (SAXS) by fitting the SAXS plot, assuming normal log distribution. The Fourier transform infrared (FTIR) spectra were recorded with a NICOLET 5700 set up in the range 400–2500 cm^−1^. The dried samples were mixed with a KBr matrix and the spectra were recorded in the transmission mode. The room temperature magnetic measurements were carried out by using a vibrating sample magnetometer (model Lakeshore 7410 VSM). The room temperature microwave resonance study was performed using a Bruker EMX-10 spectrometer with a 100 kHz modulation field. The measurements were performed using X-band (9.85 GHz) frequency in a rectangular cavity. 2,2-Diphenyl-1-picrylhydrazyl (DPPH) was used as the standard sample to validate the experimental results.

## 3. Results and discussion

### 3.1 Structural study using XRD

**Fig. 1:**
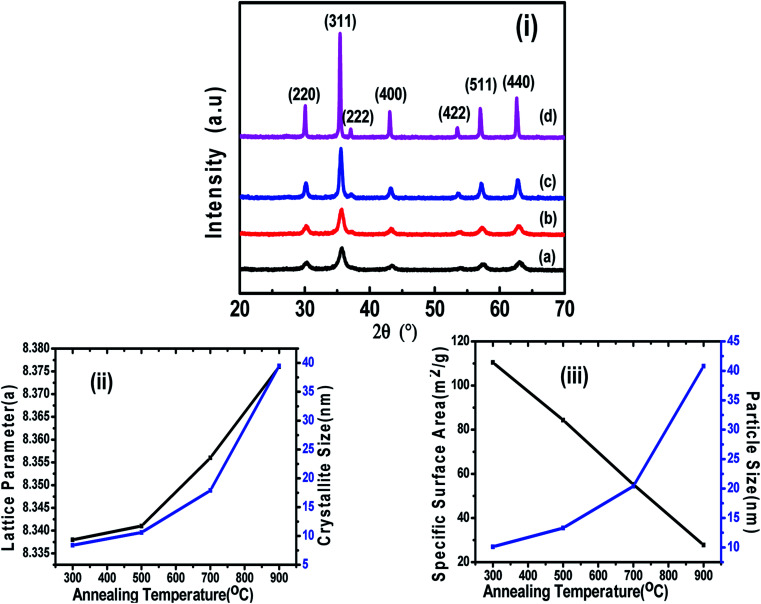
(i) XRD patterns of CoFe_2_O_4_ nanoparticles annealed at (a) 300 °C, (b) 500 °C, (c) 700 °C, and (d) 900 °C. (ii) Variations in the lattice parameter and crystallite size with annealing temperature. (iii) Variation in the specific surface area and particle size with annealing temperature.

**Fig. 2:**
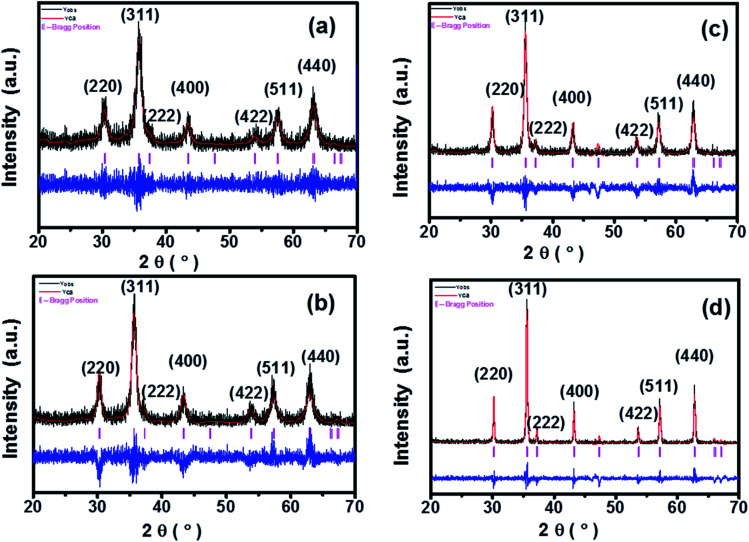
Le Bail Rietveld refinement of CoFe_2_O_4_ nanoparticles annealed at (a) 300 °C, (b) 500 °C, (c) 700 °C, and (d) 900 °C.

Fig. 1(i) shows the XRD patterns of the different annealed samples (a, b, c, d) and their Rietveld refinements are shown in Fig. 2. The Rietveld refinement was carried out using the FULLProf-suite software for the measured X-ray diffraction data of each annealed sample. All the diffraction peaks are in good agreement with the cubic spinel phase of CoFe_2_O_4_ ferrite, consistent with JCPDS card no. 22–1086.^12^ The refined profile *χ*^2^ was observed in the range 1.08 to 1.90, which is in very close proximity and evidences the existence of a single-phase cubic spinel ferrite structure with *Fd3m* (227) symmetry.^24^ The crystallite size and strain were calculated using the Debye–Scherrer (*D* = *K λ*/*β* cos *θ*) and Williamson–Hall methods 
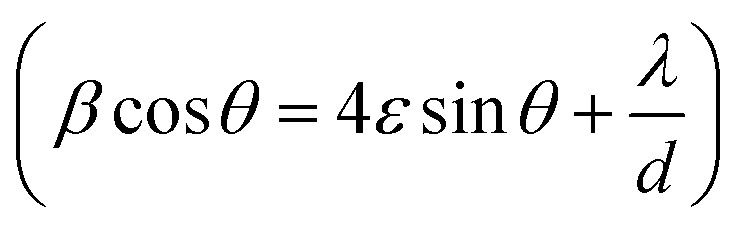
,^3,25,26^ where, *K* is Scherrer's constant (0.90), *β* = FWHM, *λ* = X-ray wavelength (*λ* = 1.5406 Å), *θ* = Bragg diffraction angle, *d* = average crystallite size, *ε* = strain.^25^ The variations in crystallite size as obtained from the Debye–Scherrer and Williamson–Hall methods and lattice parameters as obtained from Le Bail refinement are summarised in Table 1, showing the difference in the values of the lattice parameters of the annealed samples.

**Table 1:** Crystallite size, strain, average particle size, lattice parameter, X-ray density, and specific surface area of annealed CoFe_2_O_4_ nanoparticles at 300 °C, 500 °C, 700 °C and 900 °C

Sample	Crystallite size (nm) (Sherrer method)	Crystallite size (nm) (W–H method)	Strain	Average particle size (nm)	Lattice parameter (Å)	X-ray density (*d*_x_) g cm^−2^	Specific surface area “*S*” (m^2^ g^−1^)
a	8.4	5.9	0.0074	10.1	8.338	5.38	110.41
b	10.6	9	0.0051	13.3	8.341	5.35	84.32
c	17.9	14.2	0.0032	20.4	8.356	5.33	55.18
d	39.5	39	0.0024	40.8	8.376	5.30	27.74

At higher annealing temperatures, the lattice parameter and crystallite size increased, and a similar trend has also been observed in another study after annealing.^1^ The growth of the crystallite size at higher annealing temperature could be because the solid–vapour surface of the crystal was replaced by a solid–solid interface *via* diffusion, which reduced the overall surface energy during the thermal annealing and caused the expansion of the crystallite volume.^20,27^ However, the strain, X-ray density and specific surface area were decreased with the increase in annealing temperature (Table 1), which could be related to the modification of the crystal structure and particle growth.^11^ The calculated crystallite sizes of the annealed samples from both methods (Debye–Scherrer and Williamson–Hall plot), suggested the systematic increase in the crystallite size with the increase in annealing temperature, which is similar to an earlier report.^12^ We calculated the specific surface area as per the following relation:Specific surface area – *S* = 6/*d*_x_ × *D*, where *d*_x_ = X-ray density, *D* = crystalline size.

Also, the X-ray density, *ρ* of the samples was calculated from the XRD patterns using the relation, *ρ* = 8*M*/*NV*, where *M* is the molecular weight, *N* is Avogadro's number, and *V* is the cell volume.^7^Fig. 1-(ii) shows the variation in the lattice parameter and crystallite size with the increase in the annealing temperature. The variation in the specific surface area and particle size with annealing temperature is shown in Fig. 1-(iii). It can be seen clearly in Fig. 2 that the peaks became sharper for the sample with elevated annealing temperature, reflecting the increased crystallite size.

The Le Bail refinement of all the powder samples was performed using the pseudo-Voigt profile function. The values of all the refinement parameters such as the “goodness of fit” *χ*^2^, the *R* factors (RP = profile factor, RB = Bragg factor, and RF = crystallographic factor), lattice constant parameters (*a*, *b*, *c*), and the cell volumes (*V*) for all the samples were calculated, and the structural parameters obtained from profile refinement are listed in Table 2.

**Table 2:** Rietveld refinement parameters of annealed CoFe_2_O_4_ nanoparticles at 300 °C, 500 °C, 700 °C and 900 °C

Sample	χ^2^	*R*_B_	*R*_F_	*R*_WP_	*R*_exp_	*R*_P_	*V* (Å^3^)	*a* = *b* = *c* (Å)
a	1.08	0.189	0.124	46.5	44.72	48.4	577.748 (±0.392)	8.328
b	1.48	3.925	2.483	45.0	37.02	34.6	581.657 (±0.000)	8.347
c	1.79	5.660	3.696	52.0	38.87	40.0	585.413 (±0.193)	8.365
d	1.90	5.538	4.016	54.2	39.31	41.8	586.475 (±0.077)	8.370

The refined spectrum of each sample displayed a single-phase cubic spinel structure of CoFe_2_O_4_ and the goodness of fit was in the range of 1.08–1.90. The refinement was carried out by assuming the *Fd*3̄*m* symmetry (space group – 227). Further, the refinement was performed by fixing the octahedral sites (16d (1/8, 1/8, 1/8)), tetrahedral sites (8a (1/2, 1/2, 1/2)) and free oxygen (32e (*x*, *x*, *x*)) sites.^7,11^ The Rietveld refinement depicts that the Le Bail fit is optimum for extrinsic site transposal (*γ*′) equal 10% of the maxima. Moreover, the low *γ*′ value recommends the control of the size distribution of the CoFe_2_O_4_ nanoparticle samples with no or very little intrinsic variations. The lattice parameters calculated by the refinement and the position of oxygen atoms for each sample are plotted in Fig. 2(a–d). The complete process adopted for the Le Bail refinement has been described in detail in our previous work.^7^

### 3.2 FTIR analysis

**Fig. 3:**
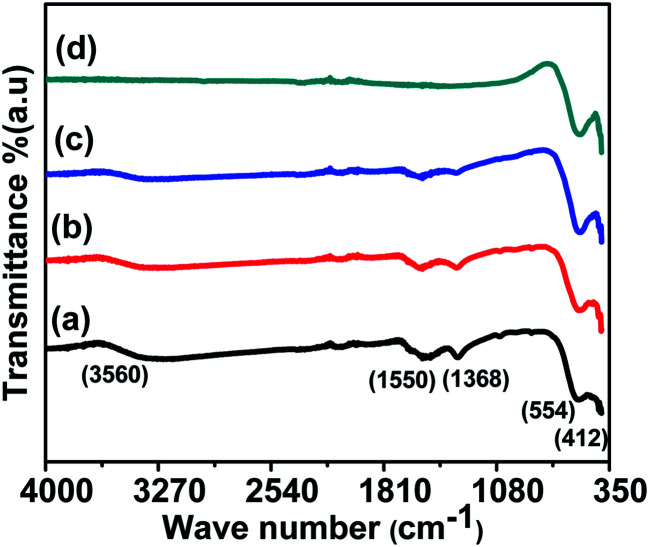
FTIR spectra of CoFe_2_O_4_ nanoparticles annealed at (a) 300 °C, (b) 500 °C, (c) 700 °C, and (d) 900 °C.

FTIR is a very useful technique for deducing molecular dynamics and it gives information not only about the positions of divalent and trivalent metal ions in the spinel lattice, but also provides their vibrational modes. Fig. 3 shows the FTIR spectra for all the samples in the range 400–4000 cm^−1^. In spinel ferrites, the IR spectral absorption bands are produced due to the vibrations of the oxygen ions with the cations present in the octahedral and tetrahedral sites in the unit cell. The characteristic region of interest for spinel ferrites is the absorptions in the 400–600 cm^−1^ range.^16^ The high-frequency band *ν*_1_ is at around 500–600 cm^−1^ for M_tetr_–O and the low-frequency band *ν*_2_ is at around 400–490 cm^−1^ for M_oct_–O. Absorption bands observed within this limit reveal the formation of single-phase spinel structure having two sub-lattices, the tetrahedral (A) site and octahedral (B) site.^12^

In this study, we observed the low-frequency band *v*_2_ at around 412 cm^−1^ and the higher frequency band *ν*_1_ at around 554 cm^−1^. The characteristic bands at 1368 cm^−1^ and 1550 cm^−1^ are due to the O–H bending peak, similar to the values obtained by Rana *et al.*^12^ Also, the O–H stretching peak was observed at 3560 cm^−1^ as shown in Fig. 3. The reduction of the O–H diffraction peak with the increase in the annealing temperature is due to the loss of residual water molecules; we have observed that with the increase in the annealing temperature to 900 °C, the peaks for water molecules were also diminished, and similar behaviour was also reported in literature.^12^ As the annealing temperature increases, the intensities of the bands corresponding to O–H stretching vibrations decrease drastically, and this might be due to the loss of residual water molecules in all of the samples.^12,16^ These band positions were found to be in agreement with the characteristic infrared absorption bands of cobalt ferrite nanocrystals because CoFe_2_O_4_ is an inverse spinel in which Co^2+^ ions occupy the octahedral sites and Fe^3+^ ions occupy the tetrahedral sites. The absorption band *ν*_1_ is caused by the stretching vibrations of the tetrahedral metal (Fe^3+^)–oxygen bond, and the absorption band *ν*_2_ is caused by the octahedral metal (Fe^3+^/Co^2+^)–oxygen vibrations in octahedral sites.^21^

### 3.3 Size distribution and morphology of samples using HRTEM and SAXS

**Fig. 4:**
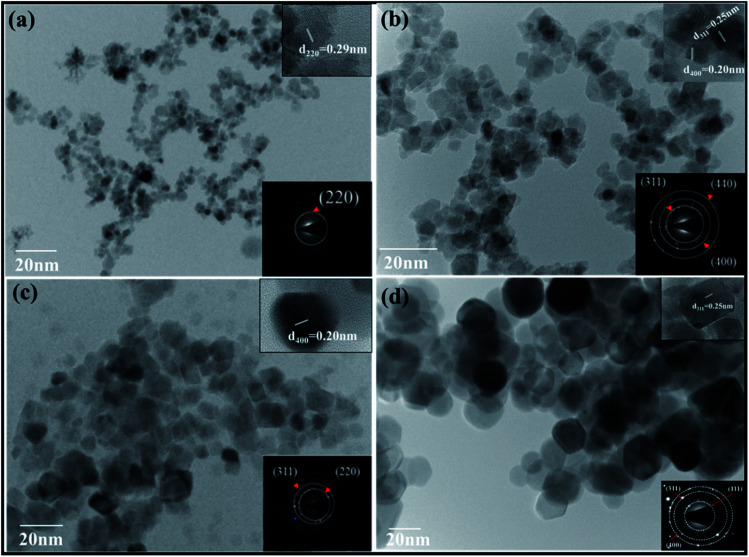
TEM images of CoFe_2_O_4_ nanoparticles annealed at (a) 300 °C, (b) 500 °C, (c) 700 °C, (d) 900 °C.

The shapes and size distributions of the annealed magnetic nanoparticles were determined by HRTEM. In all the samples, the particles were found to be spherical with uniform size distribution. The average particle size was calculated from the TEM micrograph using Image-J software; we used approximately 100 particles to plot the distribution for each sample as shown in Fig. 4. A gradual increase in the average particle size with the increase in annealing temperature was observed and is shown in Table 1. The increased average particle size could be due to the coalescence of crystallites at higher annealing temperatures.^20^ The inset in Fig. 4 shows the high-resolution TEM images of ferrite nanoparticles exhibiting interplanar spacing values with their corresponding lattice planes. The inset HRTEM micrographs show the *d*-spacing of 0.29 nm, 0.25 nm and 0.20 nm, corresponding to the (220), (311) and (400) planes, respectively. The increase in the particle size obtained from the TEM is well in agreement with the crystallite size obtained from the Williamson–Hall method. Also, the other inset in Fig. 4 for different samples shows the obtained Selected Area Electron Diffraction (SAED), confirming the polycrystalline nature of the sample and showing the various planes presented in the sample. Each plane in the SAED is shown as a white dotted circle and the corresponding planes are marked in red.

**Fig. 5:**
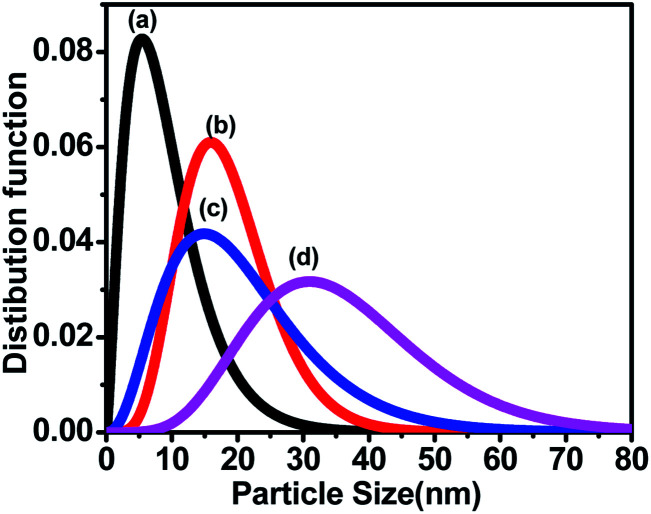
Small Angle X-ray Scattering (SAXS) shows the particle size distribution of CoFe_2_O_4_ nanoparticles annealed at (a) 300 °C, (b) 500 °C, (c) 700 °C, and (d) 900 °C.

The size distribution was also measured by Small Angle X-ray Scattering (SAXS). Fig. 5 shows the plot between the distribution function and the particle size for all the samples. The size distributions of all the samples were obtained by fitting the energy plot of the SAXS, assuming log-normal distribution and mean diameter. Fig. 5 depicts that sample ‘a’ has a narrow particle size distribution between 5–10 nm, which increased for sample ‘b’ to 5–20 nm, for sample ‘c’ to 2–35 nm and for sample ‘d’ to 10–70 nm; these are complementary to the TEM results. Sample ‘a’ depicts the left-skewed plot, which suggests that the particles in the sample are not spherical but are elongated along the *X*-axis. The SAXS plot obtained for samples ‘b’, ‘c’ and ‘d’ showed no skewness, confirming that the particles are spherical in nature.^25^

### 3.4 Static magnetic measurement using VSM

**Fig. 6:**
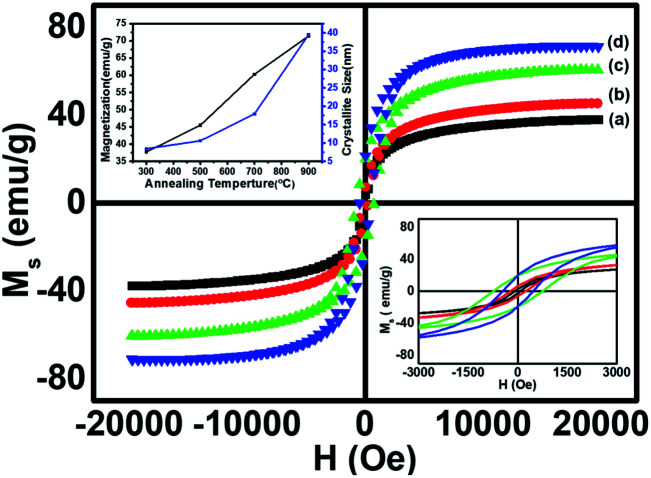
Room temperature hysteresis loops of CoFe_2_O_4_ nanoparticles annealed at (a) 300 °C, (b) 500 °C, (c) 700 °C, and (d) 900 °C.

The room temperature static magnetic measurements of all the samples were carried out with a magnetic field up to 20 kOe. The magnetic hysteresis curves confirmed the ferromagnetic behaviour in all the annealed CoFe_2_O_4_ nanoparticle samples. In general, the magnetization of the spinel ferrites depends on the distribution of the divalent and trivalent cations in the A and B sublattices of the unit cell. However, the total magnetic moment originated from the difference in the magnetic moments of the A (tetrahedral) and B (octahedral) sites, and therefore *M*_s_ depends on the distribution of the cations.^7,20^ The M–H loops of all annealed cobalt ferrite magnetic nanoparticles are shown in Fig. 6. Various magnetic parameters such as saturation magnetization (*M*_s_), coercivity (*H*_c_), remanent magnetization (*M*_r_), anisotropy constant (*k*), *etc.* were calculated and are listed in Table 3. The anisotropy constant (*k*) was calculated using equation [*k* = (*H*_c_ × *M*_s_)/0.98], where *H*_c_ is the coercivity, *M*_s_ is the saturation magnetization, *k* is the anisotropic constant.

**Table 3:** Room temperature magnetic parameters of the annealed CoFe_2_O_4_ nanoparticles at 300 °C, 500 °C, 700 °C and 900 °C

Sample	*M*_s_ (emu g^−1^)	*H*_C_ (Oe)	*M*_r_ (emu g^−1^)	*M*_r_/*M*_S_	Anisotropy constant (*k*) (erg cm^−3^)
a	37.7	82.9	2.4	0.06	0.32 × 10^4^
b	45.5	233.4	6.2	0.13	1.08 × 10^4^
c	60.3	750.9	19.2	0.31	4.62 × 10^4^
d	71.4	459.6	19.3	0.27	3.35 × 10^4^

The M–H loops revealed the transition from the single domain to the multi-domain nature with the increase in the annealing temperature from 700 to 900 °C, which led to an increase in the coercivity of sample ‘c’ and a decrease in the coercivity of sample ‘d’.^21^ The saturation magnetization (*M*_s_) increased significantly from 37.7 emu g^−1^ to 71.4 emu g^−1^ (Table 3) with the increase in annealing temperature from 300 to 900 °C, showing the enhancement of ferromagnetism at a higher annealing temperature. The increase in the *M*_s_ value for larger particle size was found at a higher annealing temperature, showing the dependence of *M*_s_ on the particle size.^12^ However, the coercivity increased gradually for the samples annealed at temperatures from 300 to 700 °C and decreased on further increase in the annealing temperature to 900 °C. This trend of change in the coercivity indicates the presence of the single domain nature in samples ‘a’, ‘b’ and ‘c’, and the multi-domain nature in sample ‘d’. At 700 °C the particle exhibited the single domain nature with the maximum value of coercivity among all the annealed samples because of the coherent rotation of spins, which is consistent with the literature.^17^ The critical average particle size for the single domain was observed around 20 nm (sample c). The increase in coercivity from sample ‘a’ to sample ‘c’ (Table 3) could be attributed to the increase in the magnetic anisotropy for sample ‘c’.^21,22^ The magnetic anisotropy and coercivity decreased on increasing the annealing temperature to 900 °C, due to the formation of the multi-domain region within the particles of sample ‘d’.^1,28^ The variation in the saturation magnetization and coercivity with particle size could also be explained on the basis of the magnetic domain structure, super-exchange interaction, particle diameter, magneto-crystalline anisotropy and shape anisotropy.^20^

Combining all the magnetic properties of the annealed CoFe_2_O_4_ nanoparticles, we can conclude that the saturation magnetization (*M*_s_) value of the annealed samples increased up to 71.4 emu g^−1^ on increasing the annealing temperature up to 900 °C, which is significantly lower than the bulk CoFe_2_O_4_ value of 90 emu g^−1^.^12^ Our results for *M*_s_ are better at the nano-level and are higher than reported by Sajjia *et al.* (62 emu g^−1^).^13^ The lower value of *M*_s_ for nanoparticles could be attributed to the finite size effect. The finite-size effect shows a non-collinearity (spin canting) in the spinel structure (tetrahedral, A site, S_A_ and octahedral, B site S_B_) that results in a decrease in the net magnetic moment.^7,27,29^ This is in accordance with the two-sublattice Néel model, defined as the difference between the moments of the A sublattice and B sublattice. The increase in the particle size favoured the increase in the collinearity of spins (S_A_ and S_B_) in the ferrimagnetic structure, which enhanced the saturation magnetization at higher annealing temperatures. The squareness ratio (*R* = *M*_r_/*M*_s_) was between 0.06–0.27, which decreased with an increase in the annealing temperature due to the decrease in the energy loss per unit cycle and its value was less than 0.5, so there was a uniaxial anisotropy according to the Stoner Wohlfarth model.^7,12^ Also, for nano-sized ferrite particles, the surface area is larger and thus the surface energy and surface tension are higher as compared to the bulk.

Generally, in the collinear ferromagnetic (FiM) structure, the value of the magnetic moment per formula unit is calculated by considering the collinear two-sublattice Néel model of ferrimagnetism. In the present investigation, the calculated moment for each of the samples from the theory does not match the experimental values obtained from VSM. This discrepancy may be due to the spin canting at the B site; thus, we considered the three-sublattice Yafet–Kittel model to calculate the spin canting (basically a lack of full alignment of spins), which was already discussed in detail in our recent publication.^7^ The canting angle *ϕ* was calculated using the following assumption: *M* = MB cos *ϕ* − MA. The calculated values of the canting angles were 39.81, 32.44, 33.63 and 33.29 for the samples a, b, c and d respectively.

**Fig. 7:**
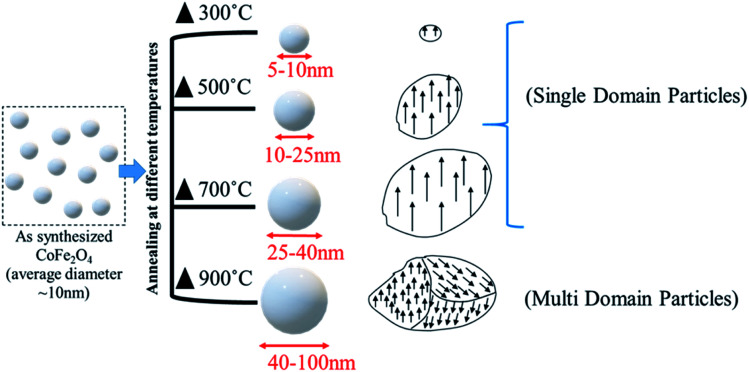
The effects of the annealing temperature on the domain structure of the nanoparticles.

The schematic representation of the annealing effects on the domain structure of the CoFe_2_O_4_ nanostructure is shown in Fig. 7. The schematic indicates that the increase in the particle size from 5–100 nm has two different domain wall structures. The annealed nanoparticles (300–700 °C) were ferromagnetic with a single-domain nature, which converted to multi-domain particles upon annealing at 900 °C.

### 3.5 Spin dynamics investigation using FMR

**Fig. 8:**
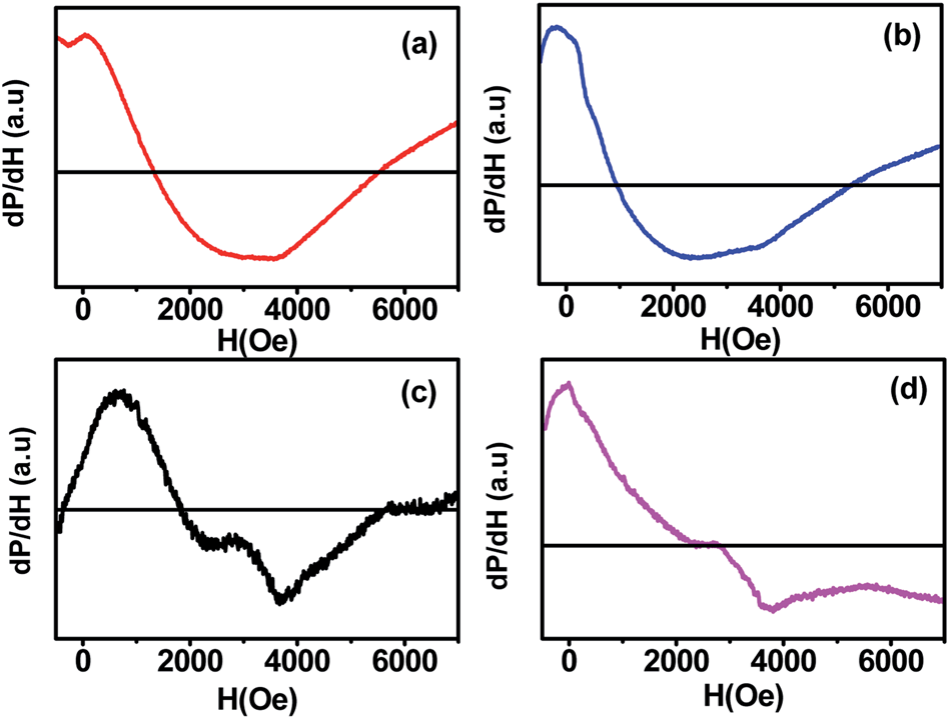
Room temperature ferromagnetic resonance spectra (FMR) of CoFe_2_O_4_ nanoparticles annealed at (a) 300 °C, (b) 500 °C, (c) 700 °C, and (d) 900 °C.

The spin dynamics of the magnetic nanoparticles have also been studied by performing ferromagnetic resonance (FMR) measurements. Fig. 8 shows the room temperature FMR spectra of samples (a, b, c, d) annealed at different temperatures. The resonance field increased with the increase in the annealing temperature, which could be due to the change in the super-exchange interactions between metallic ions through oxygen ions. Also, the lower magnetic moment of the smaller-sized sample increased the capability to attain the required splitting energy at lower magnetic field values; the sample showed a resonance field of 1057.72 G. With the increase in the annealing temperature, the resonance field first decreased to 721.54 G for sample b and then increased gradually to 1925.08 G for sample d. The FMR spectrum obtained for each sample was fitted with different fitting models (Lorentzian, pseudo-Voigt, and Gaussian) but the fitting did not converge for any of the functions due to the asymmetric behaviour of the FMR line shape. Thus, to calculate Δ*H*_PP_, we divided the curve into two parts and the peak position of each curve was taken. The difference in the x-coordinate of the peak points represents the Δ*H*_PP_, which is used to calculate the spin resonance parameters. Further, we confirmed the values from the Win Acquisition software by taking the peak position of the derivative curve, which is in close proximity. The decrease in the resonance field (*H*_r_) for sample ‘b’ can be attributed to the decrease in the line width (Δ*H*_PP_) due to the stronger super-exchange interactions between the cations through the oxygen ions (Fe_A_^3+^ − O^2−^ − Fe_B_^3+^) & (Fe_A_^3+^ − O^2−^ − Co_B_^2+^) at A-sites and B-sites.^23^ The enhancement in the super exchange-interaction also increased the *g*-value for sample ‘b’ due to the magnetic ordering within each sublattice of CoFe_2_O_4_ ferrite, which led to the rearrangement of cations at the A-site and B-site.^15^ On annealing the nanoparticles at 700 and 900 °C, the Co^2+^ ions transferred from B-sites to A-sites, which caused the reduction in the super-exchange interactions between the metallic cations of spins at A-sites and B-sites through oxygen ((Fe_A_^3+^ − O ^2−^ − Fe_B_^3+^) and (Co_A_2^+^ −O ^2−^ − Fe_B_2^+^)). The A-site is shared by both Fe^3+^ and Co^2+^ ions in these samples. Consequently, the resonance field (*H*_r_) and line width (Δ*H*_PP_) increased, whereas the *g*-value decreased (see Table 4) at higher annealing temperatures (700 and 900 °C). The reduction in the line width and enhancement in the *g*-value at higher temperature treatment of CoFe_2_O_4_ ferrite was also observed in a previous study.^10^ The increase in the peak to peak linewidth also indicated the enhancement in the dipole–dipole interactions in the CoFe_2_O_4_ nanoparticles with the increase in the annealing temperature from 500 to 900 °C.^30^

**Table 4:** Spin dynamics parameters of annealed CoFe_2_O_4_ nanoparticles at 300 °C, 500 °C, 700 °C and 900 °C

Sample Name	*H*_r_ (G)	Δ*H*_pp_ (G)	Δ*H*_1/2_ (G)	*g*-value	*N*_S_ (spin per g)
a	1057.72	3229.69	5593.98	1.8	7.6471 × 10^22^
b	721.54	2256.25	3907.93	2.0	4.8080 × 10^22^
c	1902.37	2995.08	5198.63	1.4	9.1177 × 10^22^
d	1925.08	3041.98	5268.86	1.2	10.8039 × 10^22^

The values of the spin resonance parameters are shown in Table 4. The spin concentration (*N*_S_), as well as the peak to peak line width (Δ*H*_PP_), significantly depends on the annealing temperature. The dipole–dipole interactions and linewidth increased at higher annealing temperatures, resulting in higher *N*_S_.^30^ The presence of the Co^2+^ ions in CoFe_2_O_4_ caused the high magneto-crystalline anisotropy of the material and broadened the FMR spectra.^10^ Generally, *N*_S_ is calculated by the comparison method with a standard DPPH, in which the area under the curve of DPPH (contains 1.52718 × 10^18^ spins per g) is compared with the area under the curve of the samples, provided all the conditions such as modulation amplitude, time constant and gain are the same. However, this method is not very reliable, so we calculated *N*_S_ by statistical theory, which predicts that when the line shape is Lorentzian or between Lorentzian and Gaussian. The spin concentration can be calculated by the following equation:^7,31^
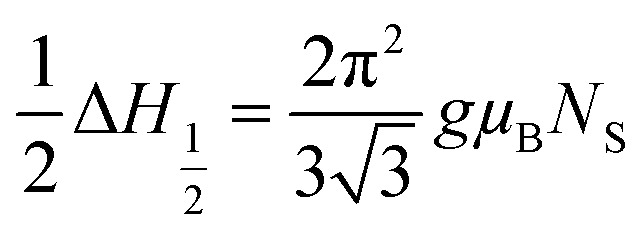

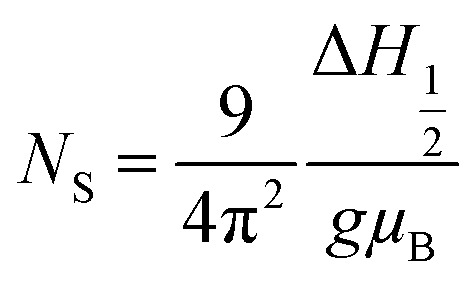
where *N*_S_ is in cm^−3^ and Δ*H*_1/2_ is the linewidth (in Gauss) at half height of the absorption peak, Δ*H*_1/2_ = √3 Δ*H*_PP_, 
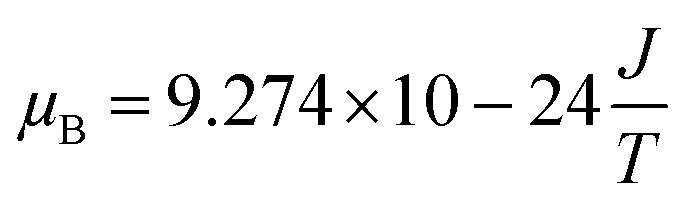
. The results obtained from the equation are given in Table 4. The asymmetric shape of the FMR spectra of samples ‘c’ and ‘d’ as shown in Fig. 8 could be related to the high anisotropy present in these samples.^32^

## 4. Conclusions

Spin resonance phenomena and theory are of key importance for various applications. The FMR technique is a unique tool for investigating the spin dynamics behaviour of magnetic materials. In this work, we have investigated the spin dynamics in CoFe_2_O_4_ samples of varying sizes by fitting the FMR spectra with various theoretical models presented in the literature. There was a systematic variation in the spin resonance parameters such as resonance field, *g*-value, spin concentration and line width. The spin resonance parameters were significantly affected by the size distribution of the particles. The resonance parameters initially decreased with the size distribution and increased after the critical size at which the material system changed from the single domain to the multi-domain. Broad FMR spectra were observed for all the samples with an asymmetric nature due to the high magneto-crystalline anisotropy. The structural and magnetic properties of annealed CoFe_2_O_4_ magnetic nanoparticles have been discussed in detail. The experimental investigation revealed that annealing significantly affects the physical and chemical properties of the CoFe_2_O_4_ but no remarkable changes were observed in the crystalline phase of the particle. From the XRD results, we can conclude that the crystallite size increased from 5.9 to 39 nm at higher temperature annealing (300–900 °C). The average particle size was increased from 10 nm to 41 nm on annealing the as-synthesized sample from 300 to 900 °C, confirmed by TEM and SAXS studies, leading to a decrease in the specific surface area of the particle. Also, the strain factor was reduced for the particles annealed at a higher temperature. The formation of magnetic nanoparticles was confirmed by the presence of the low-frequency band *ν*_2_ at around 412 cm^−1^ and the higher frequency band *ν*_1_ at around 554 cm^−1^ in the FTIR spectra. The static magnetic measurements revealed the transitions from the single domain to the multi-domain particles on annealing the sample at 900 °C. The saturation magnetization *M*_s_ value increased from 37.7 emu g^−1^ to 71.4 emu g^−1^ with the increase in the annealing temperature. The highest coercivity (750.9 Oe) and anisotropy constant (4.62 × 10^4^ erg cm^−3^) were observed for the sample annealed at 700 °C. The wide FMR spectra revealed the ferromagnetic behaviour of CoFe_2_O_4_ with a highly anisotropic nature and the resonance field shifted to a higher applied field with an increase in the annealing temperature due to the increase in the dipole–dipole interaction. The characteristic bands at around 1368 cm^−1^ and 1550 cm^−1^ were due to the O–H bond. Also, the spin concentration of the samples increased at higher annealing temperatures. The small particle size (∼10 nm) and good ferromagnetic properties present in the single-domain cobalt ferrite nanoparticles make them a promising candidate for biomedical applications to improve the quality of targeted drug delivery, magnetic resonance imaging (MRI) and hyperthermia treatment. The single-domain nanomagnetic particle having higher coercivity and moderate magnetization can be used for high-density magnetic storage applications, *i.e.* magnetic tape, hard disks, *etc.*

## Conflicts of interest

There is no conflicts of interest to declare.

## Supplementary Material
